# Obstetric triage systems: a systematic review of measurement properties (Clinimetric)

**DOI:** 10.1186/s12884-020-02974-0

**Published:** 2020-05-06

**Authors:** Asieh Moudi, Mina Iravani, Mahin Najafian, Armin Zareiyan, Arash Forouzan, Mojgan Mirghafourvand

**Affiliations:** 1 Midwifery Department, Reproductive Health Promotion Research Center, Nursing and Midwifery School, Ahvaz Jundishapur University of Medical Sciences, Ahvaz, Iran; 2 Department of Obstetrics and Gynecology, School of Medicine, Fertility Infertility and Perinatology Research Center, Ahvaz Jundishapur University of Medical Sciences, Ahvaz, Iran; 3 Public Health Nursing Department, Nursing Faculty Aja University of Medical Sciences, Tehran, Iran; 4 Department of Emergency Medicine, School of Medicine, Imam Khomeini General Hospital, Ahvaz Jundishapur University of Medical Sciences, Ahvaz, Iran; 5 Midwifery Department, Social Determinants of Health Research Center, Tabriz University of Medical sciences, Tabriz, Iran

**Keywords:** Triage, Obstetric, Pregnancy, COSMIN, Systematic review, Measurement properties

## Abstract

**Background:**

Since labor and delivery units often serve as emergency units for pregnant women, the use of obstetric triage systems with poor or inadequate quality can lead to unintended consequences such as over and under-triage and so a waste of humans and financial resources. Therefore, this systematic review was conducted to evaluate the measurement properties of obstetric triage tools.

**Methods:**

PubMed, EMBASE, and Medline were searched to identify studies in October 2018 and were updated in May 2019. The risk of bias COSMIN checklist was used to evaluate the quality of the studies. The quality of every measurement property was appraised by the update criteria of COSMIN. Evidence quality was judged using the modified GRADE approach.

**Results:**

A total of 444 studies were retrieved in initial search. Six studies evaluating 4 tools were included in this study. All the included studies reported only content validity and reliability. The quality of evidence varied from very low to moderate. The quality of content validity and reliability of the included tools was sufficient except for the reliability of the maternal-fetal triage index. The obstetric triage acuity scale (OTAS) was found to have higher reliability than other tools.

**Conclusions:**

Due to insufficient evidence, the conclusions about the quality of measurement properties of each obstetric triage tool may be uncertain. This review emphasizes the necessity for further studies with robust methodological quality on the measurement properties of obstetric triage tools.

## Background

Triage is the process of prioritizing patients based on the acuity of the problem to take the best treatment in the shortest possible time [1]. It includes brief and focused assessment and patient allocation to an acuity level, which determines the length of time a patient can safely wait for therapeutic screening examination and treatment [2]. The first time triage was used to prioritize medical care during the Napoleonic Wars in the late eighteenth century [3]. Later in the 1950s, triage was introduced in the United States as an answer to the problem of overcrowding in the emergency department (ED) of hospitals. Emergency departments needed structured triage guidelines to implement this process, so several countries have designed and presented different triage systems [4, 5]. The American College of Emergency Physicians (ACEP) also emphasizes supporting triage systems and believes that the quality, safety, and efficiency of patient care processes will be improved by implementing standardized emergency department triage acuity tools [4, 6, 7].

From 1986 to 2010, labor and delivery units that serve as emergency units for pregnant women triaged pregnant women based on standardized emergency acuity scales such as the Canadian Triage Acuity Scale (CTAS) or the Emergency Severity Index (ESI) [2, 8, 9]. Over time, studies have shown the limited applicability of these scales in obstetric triage [9–12] due to the following reasons. Firstly, obstetric triage is beyond the concise estimate and entails a thorough assessment of the mother and fetus [13]. Secondly, the acuity determinants do not reflect the variation of pregnancy manifestations or the specialized needs of obstetric patients [9]. These reasons led to the development of the first obstetric triage acuity scale in 2010 [14]. Since then, different obstetric triage acuity scales have been developed and validated, including the Florida Hospital System (FHS), Obstetric Triage Acuity Scale (OTAS), Maternal-Fetal Triage Index (MFTI), and Birmingham Symptom-Specific Obstetric Triage System (BSOTS) [6, 11, 15, 16].

However, Angelini and LaFontaine showed that the use of an acuity or risk stratification scale specific to obstetric triage is one of the components of the best obstetric triage model [2]. Nevertheless, the use of obstetric triage systems with poor or inadequate quality can lead to unintended consequences, such as “under or over-triage” and so a waste of human and financial resources. Thus assessing the measurement properties of designed tools seems essential [17, 18]. In the meantime, although it is recommended that instruments used in clinical settings be systematically evaluated for measurement properties [19], no evaluation has so far been carried out on the quality of the measurement properties for obstetric triage tools. Therefore, this systematic review was conducted to assess the quality of measurement properties of the existing obstetric triage tools based on the consensus-based standards for the selection of health measurement instruments (COSMIN) checklist. This checklist describes validity [content validity, construct validity (structural validity, hypotheses testing, cross-cultural validity), and criterion validity], reliability (internal consistency, reliability, measurement error), and responsiveness [20]. The COSMIN was developed for use in studies on the quality of patient-reported outcome measures (PROMs), but it can also be used for clinician-reported outcome measures.

## Methods

### Study type

The present study was the first systematic review conducted to evaluate the measurement properties of obstetric triage tools. This study was designed in line with the recommendations of the COSMIN initiative and reported in compliance with the Preferred Reporting Items for Systematic Reviews and Meta-Analyses Protocols (PRISMA-P) statement [20, 21].

### Eligibility criteria

Eligibility criteria for studies in this review included articles that have developed an obstetric triage tool or at least had reported one measurement property as defined in the COSMIN taxonomy related to triage in pregnant women [22]. Language and time restrictions were not considered in the search strategies. Studies were excluded if the tools were used for other purposes such as measuring outcomes or validating another tool. Editorials and conference abstracts were excluded.

### Literature search strategy

A systematic literature search was conducted between 13-23^th^October 2018 by two independent reviewers using the following electronic databases: PubMed, Medline (Ovid), and Embase. Search strategies included both free text words and subject headings and a sensitive search filter for measurement properties, which is available for PubMed, Medline, and EMBASE in the COSMIN website (See Table 1) [23, 24]. To ensure access to all available articles, a medical librarian searched the databases. The search was updated on 26 May 2019 to verify new publications. A total of 617 abstracts were retrieved as follows: PubMed = 141, Medline = 161, Embase = 308, and Grey literature = 7, of which173 duplicate abstracts were deleted.

**Table 1:** Search terms

Database	Search terms
PubMed	((((triage [tiab] OR triage [MeSH] OR acuity [tiab] OR acuity [MeSH] OR patient flow [tiab] OR patient flow [MeSH])) AND (obstetric [tiab] OR obstetric [MeSH] OR pregnancy [tiab] OR pregnancy [MeSH])) AND (scale [tiab] OR scale [MeSH] OR tool [tiab] OR tool [MeSH] OR tools [tiab] OR tools [MeSH] OR index [tiab] OR index [MeSH] OR system [tiab] OR system [MeSH])) AND provided measurement properties sensitive search filter by Terwee [23].
Embase	(‘triage’:ab,ti OR ‘triage’/exp. OR ‘acuity’:ab,ti OR ‘acuity’ OR ‘patient flow’:ab,ti OR ‘patient flow’) AND (‘pregnancy’:ab,ti OR ‘pregnancy’/exp. OR ‘obstetric’:ab,ti OR ‘obstetric’) AND (‘tool’:ab,ti OR ‘tool’/exp. OR ‘tools’:ab,ti OR ‘tools’ OR ‘scale’:ab,ti OR ‘scale’/exp. OR ‘index’:ab,ti OR ‘index’/exp. OR ‘system’:ab,ti OR ‘system’)AND Search filter for finding studies on measurement properties in EMBASE.com [23].
Medline (Ovid)	((((instrumentation or methods).sh. or (Validation Studies or Comparative Study).pt. or expPsychometrics/ or psychometr*.ti,ab. or (clinimetr* or clinometr*).tw. or outcome assessment.ti,ab. Oroutcome measure*.tw. or exp. Observer Variation/ or observer variation.ti,ab. or exp. Health Status Indicators/ or exp. Reproducibility of Results/ or reproducib*.ti,ab. or exp. Discriminant Analysis/ or (reliab* or unreliab* or valid* or coefficient or homogeneity or homogeneous or internal consistency).ti,ab. or (cronbach* and (alpha or alphas)).ti,ab. or (item and (correlation* or selection* or reduction*)).ti,ab. or (agreement or precision or imprecision or precise values or test-retest).ti,ab. or (test and retest).ti,ab. or (reliab* and (test or retest)).ti,ab. or (replicab* or repeated).mp.)and (measure or measures or findings or result or results or test or tests).ti,ab.) or (generaliza* or generalisa* or concordance).ti,ab. or (intraclass and correlation*).ti,ab. or (discriminative or factor analysis or factor analyses or dimension* or subscale*).ti,ab. or (multitrait and scaling and (analysis or analyses)).ti,ab. or (item discriminant or interscale correlation* or error or errors).ti,ab. or (variability and (analysis or values)).ti,ab. or (uncertainty and (measurement or measuring)).ti,ab. or (sensitiv* or responsive*).ti,ab. or ((minimal or minimally or clinical or clinically) and (important or significant or detectable) and (change or difference)).ti,ab. or (small* and (real or detectable) and (change or difference)).ti,ab.) and (triage or acuity or patient flow).ti,ab. and (obstetric or pregnancy).ti,ab.

### Study selection

**Fig. 1:**
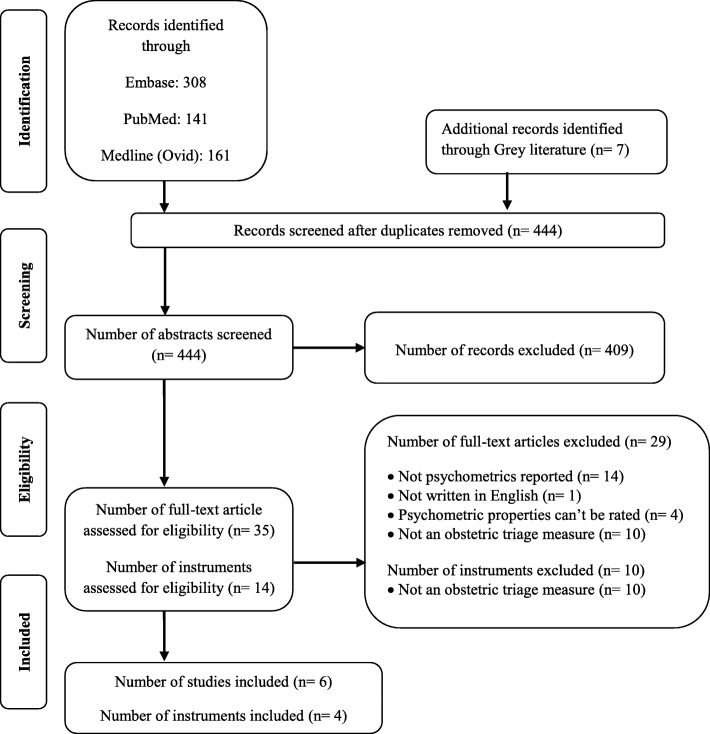
The PRISMA flow diagram for an overview of the study selected

Abstracts and full texts were independently evaluated by two of the authors (AM and MI) and were selected if they had inclusion criteria. Disagreements about choosing an article were discussed, and discordance between the two reviewers was consulted with the third author. The selection process is shown in the flowchart (Fig. 1). All references of the included articles were searched to achieve additional related studies.

### Data collection process and data extraction

The general characteristics for each study including design, the purpose, target population, and sample size of the study, and instrument characteristics, measurement properties, and methodological quality were extracted by the same two independent reviewers as previously mentioned (AM and MI).

### Methodological quality assessment of the studies

The COSMIN Risk of bias checklist was used to assess information about the measurement properties and the methodological quality of every single study. This checklist contains the 10 boxes including, PROMs development, content validity, structural validity, internal validity, cross-cultural validity (measurement invariance), reliability, measurement error, criterion validity, hypotheses testing for construct validity, and responsiveness [20, 25]. Each box of this checklist contains 3 to 35 standards referring to design requirements and preferred statistical methods [25]. Each item was rated using a 4-point rating scale (very good, adequate, doubtful, and inadequate). The overall rating of the quality of each study was determined using the lowest grade of any standard in the box was taken (i.e., “the worst score counts” principle). This quality could rate as very good, adequate, doubtful, or inadequate [20, 26] (See the attached supplement file).

### Quality of single studies on measurement properties

The result of a single study on a measurement property was rated against the updated criteria for good content validity and other measurement properties in the COSNIM. Each issue was rated as either sufficient (+), insufficient (−), or indeterminate (?) [20, 21] (See the attached supplement file). The updated criteria for measurement properties in the COSMIN are listed in Table 2.

**Table 2:** Updated criteria for good measurement properties by Terwee et al. [27] and Prinsenet al [28]

Measurement Property	Rating ^**a**^	Criteria
**Structural validity**	+	**CTT:** CFA: CFI or TLI or comparable measure > 0.95 or RMSEA< 0.06 or SRMR < 0.08^b^ **IRT/Rash:** No violation of unidimensionality^c^: CFI or TLI or comparable measure > 0.95 or RMSEA,0.06 or SRMR < 0.08 AND No violation of local independence residual correlations among thr items after controlling for the dominant factor < 0.20 or Q3’s < 0.37 AND No violation of monotonicity: adequate looking graphs or item scalability > 0.30 AND Adequate model fit: ITR:χ^2^ > 0.01 Rasch: infit and outfit mean squares ≥0.5 and ≤ 1.5 OR Z- standardized values > − 2 and < 2
	?	CTT: Not all information for “+” reported ITR/Rasch: Model fit not repored
	–	Criteria for “+” not met
**Internal Consistency**	+	At least low evidence^d^ for sufficient structural validity^e^ AND cronbach’s alpha (s) ≥ 0.70 for each unidimensional scale or subscale^f^
	?	Criteria for “at least low evidence^d^ for sufficient structural validity^e^” not met
	–	At least low evidence^d^ for sufficient structural validity^e^ AND cronbach alpha (s) < 0.70 for each unidimensional scale or subscale^f^
**Reliability**	+	ICC or weighted Kappa ≥0.70
	?	ICC or weighted Kappa not reported
	–	ICC or weighted Kappa < 0.70
**Measurement error**	+	SDC or LoA < MIC^e^
	?	MIC not defined
	–	SDC or LoA > MIC^e^
**Hypotheses testing for construct validity**	+	The result is in accordance with the hypothesis^g^
	?	No hypothesis defined (by the review team)
	–	The result is not in accordance with the hypothesis^g^
**Cross-cultural validity/ measurement invariance**	+	No important differences found between group factors (such as age, gender, language) in multiple group factor analysis OR no important DIF for group factors (McFadden’s R^2^ < 0.02)
	?	No multiple group factor analysis OR DIF analysis performed
	–	Important differences between group factors OR DIF was found
**Criterion validity**	+	Correlation with gold standard ≥0.70 OR AUC ≥ 0.70
	?	Not all information for “+” reported
	–	Correlation with gold standard < 0.70 OR AUC < 0.70
**Responsiveness**	+	The result is in accordance with the hypothesis^g^ OR AUC ≥ 0.70
	?	No hypothesis defined (by the review team)
	–	The result is not in accordance with the hypothesis^g^ OR AUC < 0.70

AUC = area under the curve, CFA = confirmatory factor analysis, CFI = comparative fit index, CTT = classical test theory, DIF = differential item functioning, ICC = intraclass correlation coefficient, IRT = item response theory, LoA = limits of agreement, MIC = minimal important change, RMSEA: Root Mean Square Error of Approximation, SEM = Standard Error of Measurement, SDC = smallest detectable change, SRMR: Standardized Root Mean Residuals, TLI = Tucker-Lewisindex

^a^ “+” = sufficient,” – “= insufficient, “?” = indeterminate

^b^ To rate the quality of the summary score, the factor structures should be equal across studies

^c^ unidimensionality refers to a factor analysis per subscale, while structural validity refers to a factor analysis of a (multidimensional) patient-reported outcome measure

^d^ As defined by grading the evidence according to the GRADE approach

^e^ This evidence may come from different studies

^f^ The criteria ‘Cronbach alpha < 0.95’ was deleted, as this is relevant in the development phase of a PROM and not when evaluating an existing PROM

^g^ The results of all studies should be taken together and it should then be decided if 75% of the results are in accordance with the hypotheses

### Summarizing the evidence and grading the quality of the evidence

The results of all available studies were qualitatively summarized to determine overall rating measurement properties as sufficient (+), insufficient (−), or inconsistent (±) based on guidelines for determining measurement properties in the COSMIN manual [20, 21]. Then, the quality of the evidence was ranked using a modified GRADE approach in a way, high, moderate, low, and very low [26].

## Results

### Systematic literature search

Six hundred and seventeen abstracts related to obstetric triage were found in search of 3 databases. After comparing the achieved results, 173 were removed due to duplicate, and the overall 444 abstracts were screened to enter this review. Abstracts were appraised, and 409 were excluded due to a lack of eligibility criteria. Thirty-five full-texts, who had studied the 14 instruments, were assessed for eligibility for the study criteria. Twenty-nine of these studies did not meet the principles for inclusion and were excluded. The reasons for eliminating were not measuring the obstetric triage (10), no reporting the psychometric properties (15), and not rating the psychometric properties (4). As a result, the measurement properties of 4 obstetric triage tools used in 6 articles were evaluated (Fig. 1).

### Included obstetric triage instruments

Table 3 displayed the characteristics of the included tools in the review. All of these were developed in the latest 8 years (since 2011). The measured construct in 3 tools is obstetric triage, and the other measures the gynecology triage in addition to the obstetric triage. The target population of all instruments is pregnant women, but the Swiss Emergency Triage Scale (SETS) include gynecology patients in addition to pregnant women [29]. All of the tools are clinician-reported outcome measures and use a dichotomous (i.e., yes or no) rating system. MFTI and OTAS tools have 5 levels, but SETS and BSOTS tools have four.

**Table 3:** Characteristics of the included instruments

Instrument (Acronym)	Construct(s)	Target population	Mode of administration	(Sub)scale(s) (number of items)	Response options	Original language	Development year	Recommended by standardization initiatives for (a specific patient population or for the construct to be measures)s	Completion time
Emergency triage scale for obstetrics and gynecology (Swiss Emergency Triage Scale = SETS) [27]	Obstetric and Gynecology triage	Pregnant women & women	Clinician-reported outcome measure	4 sub scales (NR items)	Yes/ No	English	2011	Obstetric and Gynaecology	NR^a^
Obstetric Triage Acuity Scale (OTAS) [8, 10]	Obstetric Triage	Pregnant women	Clinician-reported outcome measures	5 sub scales (37 items)	Yes/ No	English	2012	Pregnant women or obstetrical patient	NR
Birmingham symptom specific obstetric triage system (BSOTS) [16]	Obstetric Triage	Pregnant women	Clinician-reported outcome measures	4 sub scales (NR items)	Yes / No	English	2013	Pregnant women or obstetrical patient	NR
Maternal Fetal Triage Index (MFTI) [6, 7]	Obstetric Triage	Pregnant women	Clinician-reported outcome measures	5 sub scales (69 items)	Yes/ No	English	2014	Pregnant women or obstetrical patient	NR

^a^Not report

Information on the development and validation of the entered tools is described in Table 4. The development process is reported only for BSOTS and MFTI tools. The development group of BSOTS includes researchers and clinicians (obstetrician and senior midwives) who developed tools using the available evidence and consensus statements with the agreement of the local obstetric consultants. The task force that includes 6 nurses, one statistician, and one project manager, developed the MFTI based on the literature review and scenarios of actual pregnant women. The MFTI was validated by three professional groups such as physicians, certified nurse-midwives, and nurses, SETS by nurses and midwives, OTAS by nurses only, and BSOTS by midwives only. All measures demonstrated some evidence of validation through the use of a normative study sample sizes. All studies reported using professionals with work experience except OTAS studies that reported nothing about work experience. The SETS study revealed a median of 15 months of work experience for participants. About half of the participants of BSOTS worked 1–2 times a week in the obstetric triage unit, and all of the participants of MFTI had minimal 3 years of experience in triage.

**Table 4:** Description of studies for the development and validation of instrument for obstetric triage

Instrument	Reference	Purpose of study	Study population	characteristics of the study populations
**SETS**	Veit-Rubin [27]	To evaluate the inter and intra-rater reliability of SETS and to explore the factors associated with an optimal triage	*N* = 40 trained triage professionals, first phase: *n* = 22 (13 midwives and 9 nurses), second phase: *n* = 18 (10 midwives and 8 nurses) to evaluate IRR^a^ and ITR^b^ 1191 evaluation of 30 clinical vignettes	Years of health professional experience: median (IQR) = 16 (8–18), n(%): < 6 y = 4 (18.2), 6–12 y = 4 (18.2), ≥12 y = 14 (63.6) Months of experience in gynecology and obstetric: median (IQR^c^) = 42 (18–120), n (%): < 24 m = 7(31.8), 24–48 m = 4 (18.2), ≥48 m = 11 (50.0) Months of experience with the triage process: median (IQR^c^) = 15 (3–36), n (%): < 1 y = 10 (45.4), > 1 y = 12 (54.6)
**OTAS**	Smithson [11]	To test the interrater reliability and validity of OTAS and to determine the distribution of patient acuity and flow by OTAS level	*N* = 8 triage nurses to test IRR of 110 clinical Scenarios	Not reported
	Gratton [9]	To compare the inter-rater reliability (IRR^a^) in tertiary and community hospital settings and measure the intra-rater reliability (ITR^b^) of OTAS; to establish the validity of OTAS, and to present the first revision of OTAS from the national obstetrical triage working group.	*N* = 7 triage nurses to determine ITR^b^ of 110 clinical scenarios by test-retest reliability & *n* = 36 obstetrical triage nurses; London health sciences center = 8, Stratford general hospital = 11, Chatham general hospital = 7 to compare IRR^a^ in tertiary and community hospital settings	Not reported
**BSOTS**	Kenyon [16]	development, implementation and initial evaluation of BSOTS	*N* = 994 sets of maternity notes to audit before and after implementation of BSOTS by 30 clinical midwives	Age range, n (%): 20–29 y = 5 (16.7), 30–39 y = 9 (30), 40–49 y = 8 (26.7), 50–59 y = 6 (13.3), > 60 y = 2 (6.7) Years worked in midwifery, n (%): less than 1 y = 0 (0), 1–5 y = 6 (20), 6–10 y = 10 (33.3), 11–15 y = 2 (6.7), > 16 y = 12 (40) Work in triage, n (%): daily = 1 (3.3), 1–2 times/week = 13 (43.3), 1–2 times/months = 11 (36.7), 1–2 times/3 months = 5 (16.7), Never = 0 (0)
**MFTI**	Ruhl [6]	To describe the development and content validity testing of the MFTI.	*N* = 45 (round 1:11 registered nurses, 11 certified nurse-midwives, and 11 physician and round 2: 4 registered nurses, 4 certified nurse-midwives, and 4 physician)	Years caring for women in OB triage by nurses, n (%) round 1: 3–6 y = 2(18), 7–10 y = 3 (27), 11–20 y = 0(0), > 20 y = 6 (55) & round 2: 3–6 y = 0(0), 7–10 y = 0(0), 11–20 y = 0(0), > 20 y = 4 (100) Years caring for women in OB triage by physicians, n (%) round 1: 3–6 y = 2 (18), 7–10 y = 0(0), 11–20 y = 4 (36), > 20 y = 5 (45), round 2: 3–6 y = 0 (0), 7–10 y = 0 (0), 11–20 y = 1 (25), > 20 y = 3 (75) Years caring for women in OB triage by nurse-midwives, n (%), round 1: 3–6 y = 1(9), 7–10 y = 0(0), 11–20 y = 4 (36), > 20 y = 6 (55), round 2: 3–6 y = 1 (25), 7–10 y = 1 (25), 11–20 y = 0 (0), > 20 y = 2 (50)
	Ruhl [7]	To conduct interrater reliability testing of the MFTI.	*N* = 10 registered nurses for triage assessments of 211 pregnant women	Experience in obstetric triage: n(%), < 4 y = 3 (30), 5–15 y = 4 (40), 16–25 y = 1(10), 26–35 y = 2 (20) Work in triage: n(%), the day shift (7 am to 7 pm) = 5 (50), the night shift (7 pm to 7 am) = 5 (50)

^a^Inter-rater reliability

^b^Intra-rater reliability

^c^Inter-quartile range

### Measurement properties and methodological quality of the studies

Table 5 provides an overview of the quality ratings of the psychometric studies of all tools, which were evaluated against the standards of COSMIN risk of bias checklist. The development process is reported only in two BSOTS and MFTI tools. The methodological quality of tool development was rated inadequate in both studies. Content validity was done only for MFTI. According to the COSMIN checklist in content validity studies, both participants and experts should assess content relevance. In the MFTI study, the evaluation of the quality of relevance study was rated adequate according to participants, while it was rated doubtful based on the views of experts. (See the attached supplement file).

**Table 5:** Overview of the psychometric properties and methodological quality of obstetric triage tools

Instrument	Author	Year	Content validity			Structural validity	Internal consistency	Cross-cultural validity	Reliability	Measurement error	Criterion validity	Hypothesis testing
			Development	Relevance								
				participants	experts							
**SETS**	Veit-Rubin [27]	2017	NR	NR	NR	NR	NR	NR	**Adequate**	NR	NR	NR
**OTAS**	Smithson [11]	2013	NR	NR	NR	NR	NR	NR	**Adequate**	NR	NR	NR
	Gratton [9]	2016	NR	NR	NR	NR	NR	NR	**Doubtful**	NR	NR	NR
**BSOTS**	Kenyon [16]	2017	**Inadequate**	NR	NR	NR	NR	NR	**Doubtful**	NR	NR	NR
**MFTI**	Ruhl [6]	2015	**Inadequate**	**Adequate**	**Doubtful**	NR	NR	NR	NR	NR	NR	NR
	Ruhl [7]	2015	NR	NR	NR	NR	NR	NR	**Inadequate**	NR	NR	NR

*NR* not reported

Reliability property was reported for all the studies. The quality of reliability studies of SETS and Smithson’s OTAS was found adequate, those of BSOTS and Gratton’s OTAS was doubtful, and that of MFTI was inadequate. The reasons for this rating are as follows. The reliability of BSOTS and Gratton’s OTAS were calculated using un-weighted kappa, although SETS and Smithson’s OTAS used weighted kappa, the weighting scheme was not described. In the reliability study of MFTI, the reliability test conditions were not similar between raters. A rater conducted it on the patient’s bedside at arrival time, and another did it based on the patient’s electronic health record retrospectively. The first rater was inexperienced about the tool studied, while the second-rater was from the research team and familiar with the tools. Other psychometric properties were not evaluated in any of the studies. (See the attached supplement file).

### Evidence synthesis

Since according to the COSMIN guideline, if the quality of a study is rated indeterminate, it can be used from the view of reviewers in the quality rating. The quality evidence for content validity, comprehensiveness, and comprehensibility of MFTI was rated low. Because of the quality of development and content validity of examined studies were rated indeterminate, the rating was based on the view of reviewers. There was moderate-quality evidence for sufficient relevance of MFTI based on one adequate quality study of asking clinicians and a doubtful of asking experts. The insufficient reliability of MFTI was supported by very low-quality evidence (Table 6).

**Table 6:** Quality of the evidence for measurement properties of the obstetric triage tools

Measurement properties	SETS		OTAS		BSOTS		MFTI	
	Overall rating	Quality of evidence	Overall rating	Quality of evidence	Overall rating	Quality of evidence	Overall rating	Quality of evidence
	+/ - / ±^**1**^	High, moderate, low, very low	+/ - / ±	High, moderate, low, very low	+/ - / ±	High, moderate, low, very low	+/ - / ±	High, moderate, low, very low
**Content validity**	NR^a^	NR	NR	NR	NR	NR	**+**	**Low**
**Relevance**	NR	NR	NR	NR	NR	NR	**+**	**Moderate**
**Comprehensiveness**	NR	NR	NR	NR	NR	NR	**+**	**Low**
**Comprehensibility**	NR	NR	NR	NR	NR	NR	**+**	**Low**
**Structural validity**	NR	NR	NR	NR	NR	NR	NR	NR
**Internal consistency**	NR	NR	NR	NR	NR	NR	NR	NR
**Cross-cultural validity**	NR	NR	NR	NR	NR	NR	NR	NR
**Reliability**	**+**	**Low**	**+**	**Moderate**	**+**	**Low**	**–**	**Very low**
**Measurement error**	NR	NR	NR	NR	NR	NR	NR	NR
**Criterion validity**	NR	NR	NR	NR	NR	NR	NR	NR
**Construct validity**	NR	NR	NR	NR	NR	NR	NR	NR
**Responsiveness**	NR	NR	NR	NR	NR	NR	NR	NR

^a^: Not reported

(+): sufficient, (−): insufficient, (±): inconsistent

The quality of evidence reliability based on a sufficient rate for OTAS was moderate, but it was low for SETS and BSOTS. OTAS proved to be the only tool with sufficient reliability (moderate-quality evidence).

## Discussion

This systematic review was conducted to identify and evaluate the quality of the measurement properties of obstetric triage tools. Four tools were identified that evaluated obstetric triage fitting the definition used in this review. Three out of these tools were mainly developed for the pregnant population, and one was for both pregnant women and gynecology patients.

The COSMIN taxonomy was applied to guide a comprehensive summary of the measurement properties tools. This taxonomy consists of measurement properties including, internal consistency, reliability, measurement error, content (face validity), construct (hypotheses testing, structural, and cross-cultural validity), and criterion validity, and responsiveness. Since the obstetric triage tool is a formative model, the two measurement properties of internal consistency and structural validity are not relevant [20], but it is expected that other mentioned properties be tested for it. Each included study addressed just one measurement property, and overall, all of the included studies have reported only content validity and reliability. These results suggest that the current knowledge about the measurement properties of obstetric triage tools is uncertain.

### Quality of the studies using the COSMIN taxonomy

The COSMIN Risk of bias checklist provides standards about the quality of the studies that examine the measurement properties [20, 25]. The overall quality of the studies was mostly (66.7%) from inadequate to doubtful. The common reasons for these COSMIN ratings were insufficient sample size, failure to report details of study methodology, lack of clarity in reporting of statistical analysis, and lack similarity of test conditions for the measurement. Some existing deficiencies may be readily improved via more detailed reporting in future studies. Given that the critical appraisal of studies is done based on what was reported in the articles, the COSMIN checklist must be considered in future studies.

Content validity is one of the most fundamental features of measurement, and lack of its test can lead to errors in clinical judgment or inaccurate interpretation of assessment results by practitioners [30]. In this review, MFTI is the only obstetric triage tool whose content validity was assessed. Ruhl et al. used the Delphi method for content validation of this tool, which has been introduced as the best method for content validation of triage tools [6, 31]. However, the rating of study quality for content validity of MFTI was inadequate for tool development and doubtful to adequate for study relevance. It seems the MFTI may need a modification of the measures to improve evidence. The OTAS, SETS, and BSOTS did not provide any evidence of content validity, highlighting a need for further research on all of the measurement properties of these tools.

Reliability was reported for all measures with variability in the quality of studies ranging from inadequate (MFTI), doubtful (Gratton’s OTAS, BSOTS), to adequate (Smithson’s OTAS, SETS). Three assessments (MFTI, BSOTS, and Smithson’s OTAS) reported inter-rater reliability but did not examine intra-rater. The other two (i.e., SETS and Gratton’s OTAS) conducted both inter and intra-rater reliability. Statistical analysis identified as optimal (ICC or weighted kappa) was used in three studies (MFTI, SETS, and Smithson’s OTAS). As previously mentioned, the reliability study of MFTI, test conditions were not similar for the measurements.

### Overall quality of measurement properties and evidence

The findings showed that the quality of evidence of measurement properties for obstetric triage tools varied from very low to moderate. All obstetric triage tools showed sufficient measurement properties except for the reliability of MFTI.

To measure obstetric triage, the MFTI displayed moderate-quality evidence for sufficient relevance. The evidence quality of its content validity, comprehensiveness, and comprehensibility was low. The missing data for comprehensiveness and comprehensibility subscales was the reason for low overall quality score for content validity of MFTI. Given that the content validity is the first measurement property which to be considered for selecting a tool, the low evidence quality underpinning the content validity of MFTI as the recommended tool by ACOG to measure obstetric triage is worrisome. The failure to report evidence does not mean that the assessment is not valid. Thus, it is recommended that future studies evaluate measurement properties based on the recently developed COSMIN standards.

Sufficient reliability with moderate-quality evidence was observed for OTAS, while sufficient findings based on low-quality evidence were found for SETS and BSOTS. Insufficient results with very low-quality evidence were reported for the reliability of MFTI. Given the limited number of studies on obstetric triage tools and the overall ratings from very low to moderate on their measurement properties, it is evident that more research is needed in the area of obstetric triage.

### Strengths and limitations

There are several strengths in this study. The most important of these is using the most recent version of the COSMIN taxonomy to select and evaluate appropriate measurement properties. Using the checklist cause that the methodology quality of each study is individually accounted for when determining the quality of evidence, and interpreting the results. In addition to the changes made to it, this checklist is appropriate for evaluating clinician-reported outcome measures [32]. The second strength of this study is using highly sensitive validated search filters for finding studies on measurement properties that are available in the COSMIN website for PubMed, EMBASE, and Medline (Ovid). Thirdly, this study is the first systematic review that has been conducted on the measurement properties of obstetric triage tools. Fourthly, language and time restrictions were not considered in the search strategies. Lastly, the multidisciplinary presence of people with relevant expertise in the research team is another strength of this study.

The limitations of this study are; the first, the search was conducted in just 3 databases including EMBASE, PubMed, and Medline (through Ovid) because only these highly sensitive validated other databases. Second, grey literature such as conference abstracts was excluded, which may have contributed to selection bias. Finally, the number of included studies in the systematic review was limited, and the conclusion was unclear accordingly, so more studies are recommended to be conducted about the measurement properties of obstetric triage tools.

## Conclusion

This systematic review presents an in-depth insight into the current evidence about the measurement properties of obstetric triage tools. There are many knowledge gaps about the validity (i.e., content, construct, and criterion), reliability (measurement error), and responsiveness of obstetric triage tools. The quality of the majority of studies in terms of reliability ranged from doubtful to adequate. Sufficient reliability with overall ratings ranging from very low to moderate into quality evidence was observed for the majority of scales. According to available evidence, OTAS has higher reliability than other tools. However, due to insufficient evidence, the conclusions about the measurement properties’ quality of each of the obstetric triage tools may be uncertain. Therefore, this review emphasizes the necessity for further studies on the measurement properties of obstetric triage tools.

## Supplementary information

**Additional file 1.**

## Data Availability

All relevant data are given within the manuscript and the supplementary files.

## Abbreviations

COSMIN: Consensus-based standards for the selection of health measurement instruments
GRADE: Grading of Recommendations, Assessment, Development and Evaluations
OTAS: Obstetric triage acuity scale
ED: Emergency department
ACEP: American College of Emergency Physicians
CTAS: Canadian Triage Acuity Scale
ESI: Emergency Severity Index
FHS: Florida Hospital System
MFTI: Maternal-Fetal Triage Index
BSOTS: Birmingham Symptom-Specific Obstetric Triage System
PRISMA-P: Preferred Reporting Items for Systematic Reviews and Meta-Analyses Protocols
PROMs: Patient-reported outcome measures
SETS: Swiss emergency triage scale
ICC: Intra-class Correlation Coefficient
ACOG: American College of Obstetricians and Gynecologists
